# Random Splicing of Several Exons Caused by a Single Base Change in the Target Exon of CRISPR/Cas9 Mediated Gene Knockout

**DOI:** 10.3390/cells5040045

**Published:** 2016-12-14

**Authors:** Marcel Kapahnke, Antje Banning, Ritva Tikkanen

**Affiliations:** Institute of Biochemistry, Medical Faculty, University of Giessen, Friedrichstrasse 24, D-35392 Giessen, Germany; Marcel.Kapahnke@med.uni-giessen.de (M.K.); Antje.Banning@biochemie.med.uni-giessen.de (A.B.)

**Keywords:** RNA splicing, CRISPR, Cas9, genome editing, flotillin

## Abstract

The clustered regularly interspaced short palindromic repeats (CRISPR)-associated sequence 9 (CRISPR/Cas9) system is widely used for genome editing purposes as it facilitates an efficient knockout of a specific gene in, e.g. cultured cells. Targeted double-strand breaks are introduced to the target sequence of the guide RNAs, which activates the cellular DNA repair mechanism for non-homologous-end-joining, resulting in unprecise repair and introduction of small deletions or insertions. Due to this, sequence alterations in the coding region of the target gene frequently cause frame-shift mutations, facilitating degradation of the mRNA. We here show that such CRISPR/Cas9-mediated alterations in the target exon may also result in altered splicing of the respective pre-mRNA, most likely due to mutations of splice-regulatory sequences. Using the human *FLOT-1* gene as an example, we demonstrate that such altered splicing products also give rise to aberrant protein products. These may potentially function as dominant-negative proteins and thus interfere with the interpretation of the data generated with these cell lines. Since most researchers only control the consequences of CRISPR knockout at genomic and protein level, our data should encourage to also check the alterations at the mRNA level.

## 1. Introduction

The clustered regularly interspaced short palindromic repeats (CRISPR)-associated sequence 9 (CRISPR/Cas9) system has become the tool of choice for targeted gene disruption and the analysis of gene and protein function in cultured mammalian cells [1]. The CRISPR/Cas9 system requires the nuclease Cas9 and a guide RNA (gRNA) to introduce targeted double-strand breaks within DNA. In general, DNA double-strand breaks activate the cellular DNA non-homologous-end-joining (NHEJ) repair program, which frequently leads to the introduction of so-called indels (insertions/deletions), and hence to a frameshift mutation or an in-frame amino acid insertion or deletion. When located within the coding region of a gene, the presence of premature stop-codons can lead to the “knockout” of the gene of interest, especially when located within one of the first permanent exons.

For screening purposes, transfected cells are diluted to obtain single-cell clones whose changes within the DNA are identical and should exhibit the same phenotype, i.e., complete absence of expression of the protein of interest. It is considered as sufficient to validate the presence of a frameshift mutation in the putative knockout clone by sequencing of the genomic DNA, followed by assessment of protein expression and/or functional testing [2]. However, not all indels necessarily lead to premature stop codons. They might also interfere with hnRNA splicing if sequences that regulate splicing are altered. Therefore, a single indel mutation might theoretically give rise to different mRNA products within a clonal cell population. This would, instead of a complete protein knockout, result in the production of altered proteins with unpredictable functional consequences, as these proteins might, for example, gain dominant negative functions.

Flotillin-1 and -2 are ubiquitously expressed, highly conserved proteins that localize into specific cholesterol-rich microdomains (membrane rafts). Functionally, flotillins are associated with various signaling pathways and have been found to be overexpressed in many types of cancer (reviewed in [3]). Flotillin-1 is an important regulator of MAP kinase (mitogen-activated protein kinase) signaling, and it interacts with several components of the MAPK pathway [4]. Thus, flotillin-1 is required for a proper EGFR activation and downstream signaling [5]. Interestingly, flotillins not only regulate MAP kinase signaling, but they themselves are target genes of EGFR/MAP kinase mediated transcriptional regulation [6,7]. The flotillin-1 gene is located on chromosome 6 and contains 13 exons, encoding a protein with 427 residues [8].

Until recently, we have investigated the function of flotillins using siRNA or shRNA mediated knockdown. However, these methods have the disadvantage that the cells still show a varying degree of residual flotillin expression that may mask the functional consequences of loss of flotillin expression [5,9,10]. In the present study, we have used the CRISPR/Cas9 method to knock out the *FLOT1* gene in HeLa cells to obtain full genetic ablation of flotillin-1 expression. Analysis of the obtained single-cell clones by Western Blot revealed a successful *FLOT1* knockout, and the genomic changes observed were indel mutations, as expected. However, we also analyzed the consequences of the genomic changes at the mRNA level. We here show that minor genomic indel mutations caused by gRNA-mediated knockout may result in radically altered or randomized exon splicing of the respective hnRNA. These mRNA products in turn may bear the risk of giving rise to functionally altered protein products. Thus, the results of our study stress the importance of analysis of the consequences also at the mRNA and translational level.

## 2. Materials and Methods

### 2.1. CRISPR/Cas9 Plasmids for FLOT1 and AGA

Five different mammalian Cas9 genome editing constructs encoding gRNAs for *FLOT1* were obtained from Horizon Discovery (Cambridge, UK). The vector backbone for all constructs was pD1301-AD. Guide RNA sequences are shown in Table 1.

For knocking out the expression of aspartylglucosaminidase (*AGA*) in HEK293T cells, gRNAs were designed with the “optimized CRISPR design” tool (www.crisp.mit.edu) and cloned into PX459 (Addgene #48139) [2]. Transfection and cultivation of single-cell clones was performed as described for *FLOT1*. For the screening of single-cell clones, AGA enzyme activity was measured as described [11]. The gRNA sequence used was 5’-CACCGATACCCTCCAATAATTTGGC-3’.

### 2.2. Cell Culture and Transfections

HeLa (human cervix carcinoma cells) and HEK 293T (human embryonic kidney) cells were cultured in Dulbecco’s modified Eagle’s medium (DMEM, Gibco, Thermo Fisher Scientific, Karlsruhe, Germany) with high glucose, supplemented with 10% fetal calf serum (FCS, Gibco), 100 U/mL Penicillin and 100 µg/mL Streptomycin (Sigma-Aldrich, Taufkirchen, Germany) at 8% CO_2_ and 37 °C.

Twenty-four hours prior to transfection cells were seeded onto 12-well plates. For transfections, 1 µg genome editing plasmid was transfected using MACSfectin™ (Miltenyi Biotec, Bergisch Gladbach, Germany) according to the manufacturer’s protocol. After 24 h, cells were counted and seeded into 96 well plates (1 cell/well). Wells containing single-cell clones were expanded and used for further analysis.

### 2.3. Analysis of Single-Cell Clones

Flotillin-1 expression in single-cell clones was analyzed by Western Blot using a monoclonal mouse anti-flotillin-1 antibody that recognizes an epitope in the C-terminal half of flotillin-1 (BD Transduction Laboratories, Franklin Lakes, NJ, USA). A mouse monoclonal antibody against GAPDH was obtained from Abcam (Cambridge, UK) and served as a loading control. Genomic DNA and total RNA were isolated with peqGold Trifast (Peqlab, Erlangen, Germany) from two *FLOT1* knockout HeLa single-cell clones or from *AGA* HEK 293T clones. RNA was reverse transcribed with Superscript^®^ III reverse transcriptase using oligo(dT)_18_ primers (New England Biolabs, Frankfurt, Germany). *FLOT1* cDNA covering the coding region and 570 bp of genomic DNA surrounding the gRNA target site were PCR-amplified out of cDNA or genomic DNA, respectively, using Q5^®^ High Fidelity DNA polymerase (NEB, Frankfurt, Germany). The resulting products were cloned using the restriction sites contained within the primers (KpnI and EcoRI) into pEGFP-N1 (Clontech, Takara, Saint-Germain-en-Laye, France). *AGA* cDNA was PCR amplified and cloned into pcDNA3 (Invitrogen, Waltham, MA, USA) with BamHI and XhoI. The primer sequences are shown in Table 2.

Several clones derived from each single-cell population were sequenced, and the sequences were analyzed with Blast^®^ (NCBI, Bethesda, MD, USA) using *FLOT1* genomic NC_000006.12 (region: complement of 30727709-30742851) and transcript NM_005803.3 sequences or *AGA* cDNA sequence X55330.1 as reference. The ExPasy tool “Translate” was used to predict the consequences of the observed nucleotide changes.

### 2.4. Quantitative Real-Time PCR

For qPCRs, 3 µg total RNA was reverse transcribed with 150 fmol oligo(dT) primers and the M-MuLV reverse transcriptase (NEB) in a total volume of 45 µL. Real-time quantitative PCRs were performed using the CFX Connect Real-Time PCR Detection System (Bio-Rad, Munich, Germany). Annealing temperature was 60 °C for all primers. The reactions were done as duplicates with 0.8 µL of 5-fold diluted cDNA in a total volume of 10 µL using iTaqTMUniversal SYBR Green Supermix (Bio-Rad). PCR products were quantified with the ΔCt-method. For normalization, the geometric mean of the reference genes Rpl13a, B2M and Ywhaz was used. The primer sequences are shown in Table 3.

## 3. Results

**Figure 1:**
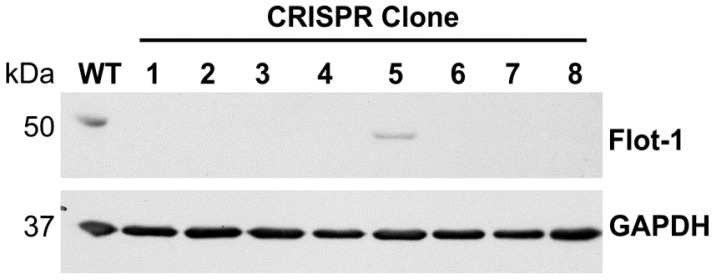
Preliminary Western Blot analysis of Hela flotillin-1 knockout clones. GAPDH was used as a loading control.

Flotillin-1 knockout HeLa cells were produced by transfecting the cells with gRNA CRISPR/Cas9 plasmids targeting the human *FLOT1* gene. Five gRNA sequences were tested (data not shown), of which gRNA 165723 showed superior knockout efficiency and was chosen for further analysis. HeLa cells were transfected with this gRNA, and single-cell clones originating from two separate experiments were selected by limiting dilution. The clones were tested by means of Western Blot (Figure 1), and clones showing a complete lack of detectable full-length flotillin-1 protein were expanded and cultured further.

**Figure 2:**
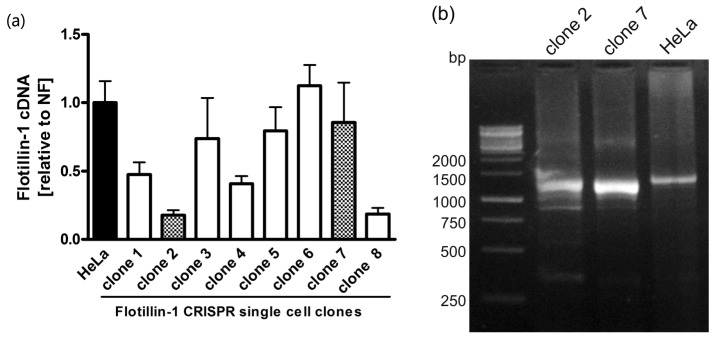
Quantitative real-time PCR analysis and PCR amplification of the cDNA of WT and flotillin-1 knockout HeLa clones. (**a**) qPCR analysis. Bar corresponding to wildtype HeLa cells is shown in black, shaded bars show the clones 2 and 7 that were chosen for further analysis. (**b**) cDNAs from clones 2 and 7 were subjected to PCR amplification with primers flanking the coding region of flotillin-1.

To check the effect of *FLOT1* gene knockout on the abundance of the respective mRNA, qPCR was performed. As shown in Figure 2, the single-cell clones showed varying amounts of flotillin-1 mRNA, ranging from wild-type levels to about 20%.

**Figure 3:**
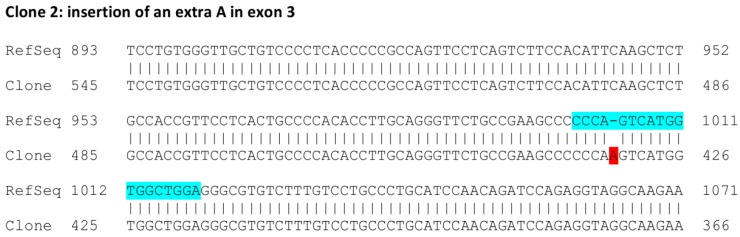
Sequence analysis of the genomic DNA of *FLOT1* knockout clone 2. A genomic region of 570 bp comprising exon 3 and the flanking intronic regions was PCR amplified, cloned into a vector, and six clones were sequenced. The numbering of the reference sequence (upper row) corresponds to the human *FLOT1* reference gene sequence (NC_000006.12, region 30.727.709-30.742.851, complementary strand). The gRNA sequence is highlighted in light blue, the insertion of an A with red.

**Figure 4:**
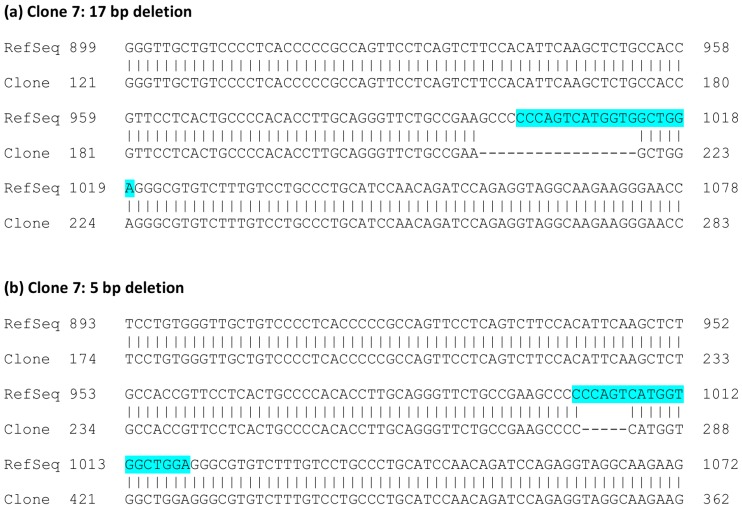
Sequence analysis of the genomic DNA of *FLOT1* knockout clone 7. A genomic region of 570 bp comprising exon 3 and the flanking intronic regions was PCR amplified, cloned into a vector, and six plasmid clones were sequenced. The numbering of the reference sequence (upper row) corresponds to the human *FLOT1* reference gene sequence (NC_000006.12, region 30.727.709-30.742.851, complementary strand). The gRNA sequence is highlighted in light blue. (**a**) Clones containing the 17 bp deletion; (**b**) clones containing the 5 bp deletion.

To analyze the consequences of gRNA-mediated *FLOT1* knockout at genomic and mRNA level in detail, we chose clones 2 and 7, which originate from separate transfections, for further analysis. The *FLOT1* gene resides on human chromosome 6. Since HeLa cells are polyploid and exhibit three copies of chromosome 6, direct genomic sequencing was not feasible. Thus, the genomic region around the targeted exon 3 was PCR amplified with primers that reside within the flanking introns and contain suitable restriction sites, and the resulting PCR products were cloned into a plasmid and sequenced. Sequence analysis of several genomic clones (Figure 3) showed that clone 2 always exhibited the same alteration: an insertion of an extra base A in exon 3. The analyzed sequence also covers the flanking intron borders on both sides of exon 3. In the case of clone 7, deletions of 5 and 17 bp residing within the gRNA target sequence were observed (Figure 4). No other sequence variants within the analyzed region were identified.

**Figure 5:**
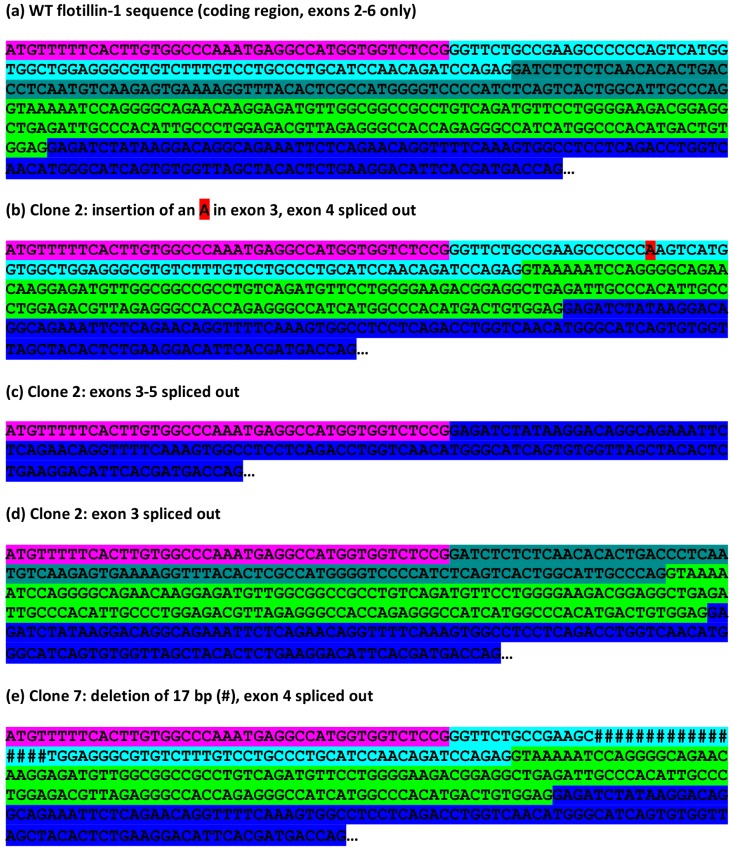
Consequences of the CRISPR/Cas9-mediated *FLOT1* knockout in HeLa cells at mRNA level. Total RNA was isolated from the WT and knockout HeLa clones and reverse-transcribed. The coding region of flotillin-1 was amplified with specific primers, cloned into a vector, and six plasmid clones were sequenced. (**a**) WT human flotillin-1 coding region (exons 2–6 only) with color-coded exons (pink: exon 2 beginning from the ATG start codon); (**b**) clone 2 with insertion of an A in exon 3 and exon 4 spliced out; (**c**) clone 2: exons 3–5 spliced out; (**d**) clone 2: exon 3 spliced out; (**e**) clone 7: deletion of 17 nucleotides (marked with #), exon 4 spliced out.

To study the effects at the mRNA level, cDNA was prepared from total RNA of WT and knockout HeLa cells and PCR amplified using primers specific for the coding region of flotillin-1 (Figure 2b). The resulting fragments were cloned into a plasmid. The mRNA sequences obtained from the WT HeLa cells are identical to the *FLOT1* reference sequence (Figure 5a). In the case of HeLa clone 2, we were expecting on the basis of the genomic alteration (insertion of an A) to detect mRNA clones that show the insertion and thus result in an early frame-shift and non-existent flotillin-1 protein. However, clones corresponding to such an mRNA species were never observed. Instead, all sequenced clones corresponded to mRNA species that had undergone differential splicing of exons 3–5 (Figure 5). Figure 5a shows the wild type flotillin-1 cDNA sequence with color coding of the exons. In several clones, exon 3 contained the expected A insertion, corresponding to the genomic defect, together with a deletion of exon 4 (Figure 5b). In addition, clones deleted for exons 3, 4 and 5 (Figure 5c) or exon 3 (Figure 5d) were observed. These data suggest that the single nucleotide insertion within exon 3 profoundly alters the splicing of several exons in *FLOT1* mRNA. However, the splicing events appear to take place at the correct exon-intron borders.

The HeLa knockout clone 7, which contained genomic deletions within the gRNA target sequence, exhibited cDNA clones corresponding to mRNA species with the observed 17 bp deletion combined with a deletion of exon 4 (Figure 5e). However, cDNA clones exhibiting the 5 bp deletion were never observed, implicating that such mRNA species may be unstable.

Since it is possible that such aberrant splicing might be specific for flotillin-1 or HeLa cells, we analyzed an independent gene, human *AGA* (aspartylglucosaminidase) which was knocked out in HEK 293T cells. Similarly to CRISPR/Cas9 mediated flotillin-1 knockout, a gRNA residing in *AGA* exon 6 resulted in altered splicing patterns of exons 2–6 (Supplementary Materials Figure S1). These data show that the aberrant splicing induced by gRNAs is neither flotillin nor cell line specific.

**Figure 6:**
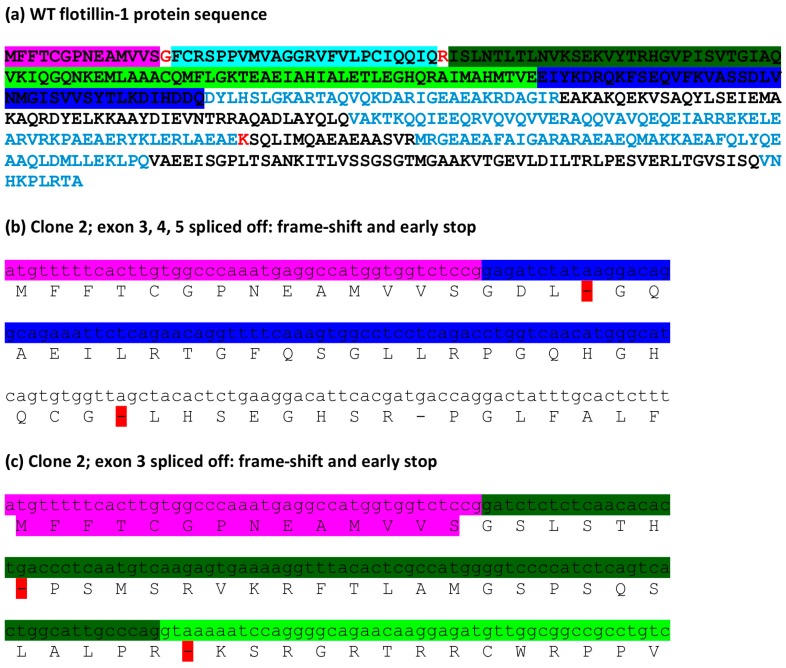
Predicted consequences of the changes in *FLOT1* mRNA sequences at the protein level: frame shift and early stop of translation. ExPasy Translate tool was used to convert the sequencing data to protein sequences. (**a**) WT flotillin-1 protein sequence with the same exon color coding as in Figure 5. The codons for the residues marked in red are compiled from the flanking exons; (**b**) clone 2: removal of exons 3–5 results in a frame shift and early stop; as does (**c**) the removal of exon 3.

To check the possible consequences of the observed splicing errors at the protein level, the cDNA sequences were translated *in silico*. Figure 6a shows the full-length human flotillin-1 protein sequence. Deletions of exons 3–5 or exon 3 result in an early stop codon after less than 20 residues from the N-terminus (Figure 6b,c). These changes thus do not produce any functional protein product and can be expected to be true knockouts.

**Figure 7:**
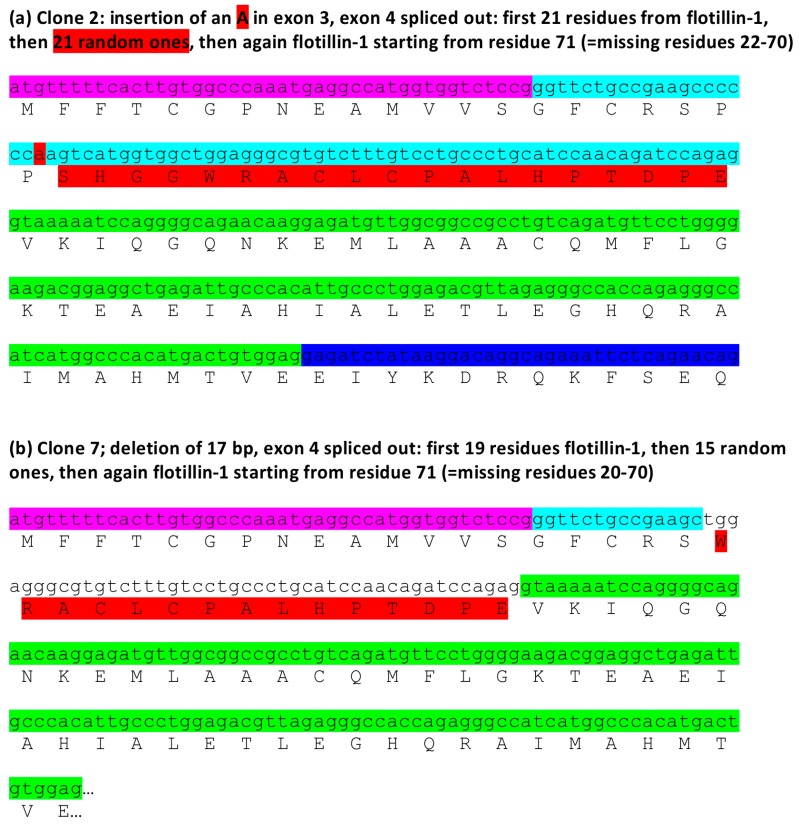
Predicted consequences of the changes in *FLOT1* mRNA sequences at the protein level: putative residual protein products. ExPasy Translate tool was used to convert the sequencing data to protein sequences. Color coding as in Figure 6. (**a**) Clone 2: insertion of an A combined with removal of exon 4 results in a putative protein product that contains the first 21 residues of flotillin-1, then 21 random ones, after which the flotillin-1 sequence continues from residue 71. (**b**) Clone 7: deletion of 17 bp, combined with the removal of exon 4 produces a putative protein containing the first 19 residues flotillin-1, then 15 random ones, after which the flotillin-1 sequence continues from residue 71.

In contrast to the above mentioned errors, the insertion of one base (A) within exon 3 combined with exon 4 removal is predicted to produce a protein product that contains 21 amino terminal residues of flotillin-1, followed by 21 random amino acids and then again flotillin-1 protein sequence starting from residue 71 (Figure 7a). This is due to the fact that although the insertion of a single A in exon 3 produces a frame shift, this is again compensated by the removal of exon 4, so that the reading frame from exon 5 onwards is corrected. Interestingly, also the 17 base deletion combined with exon 4 removal should produce a similarly altered protein product: 19 amino terminal residues of flotillin-1, 15 random ones, followed by the flotillin-1 sequence starting with residue 71 (Figure 7b). These data suggest that these mRNA species could give rise to truncated proteins that may even act in a dominant negative fashion.

We performed a similar analysis for the mRNA species detected in *AGA* gene knockout cells (Supplementary Materials Figure S2). Also in this case, the splicing defect removing exons 2–6 resulted in an early STOP codon and a severely truncated protein. However, removal of exons 3–6, which does not result in a frameshift, could produce a truncated protein product in which residues 95–232 are missing. This change would be predicted to result in a truncated protein that is most likely highly unstable, in accordance with the effects described for *AGA* gene mutations that result in aspartylglucosaminuria [12].

**Figure 8:**
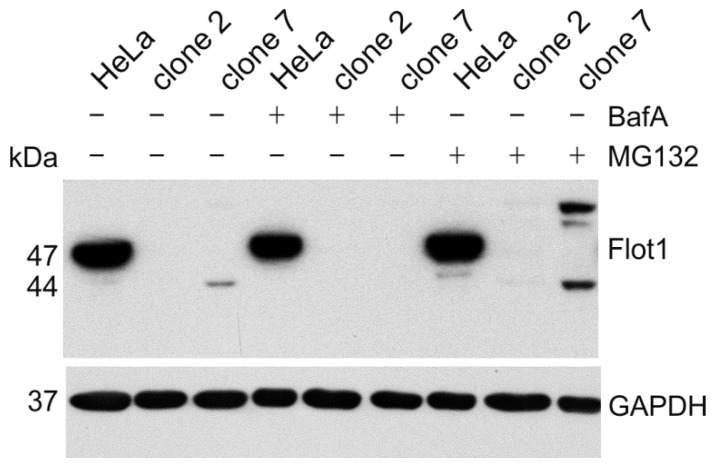
Western Blot analysis of cell lysates of clone 2 and clone 7. HeLa cells and clones 2 and 7 were treated for 24 h with 50 nM bafilomycin A (BafA) or 10 µM MG132 and blotted with anti-flotillin-1 antibody.

To demonstrate that the aberrant flotillin-1 protein products are expressed, we performed Western Blot analysis of the cell lysates (Figure 8). Since the aberrant proteins may be unstable, we used inhibitors of lysosomal acidification (bafilomycin A) or the proteasome (MG132) to inhibit protein degradation. In clone 2, we were not able to detect any protein fragments for flotillin-1. However, in clone 7, we observed after a prolonged exposure of the blot a truncated flotillin-1 protein with a molecular mass of about 44 kDa, which is well in accordance with the calculated 43.5 kDa for the aberrant protein depicted in Figure 7b. In addition, a protein product with a higher molecular mass than the WT flotillin-1 protein was observed. This signal probably originates from the ubiquitinated protein that remains undegraded upon inhibition of the proteasome. Unfortunately, a similar analysis could not be performed for the *AGA* knockouts, as none of the available antibodies is sensitive enough to detect endogenous AGA protein fragments if they are poorly expressed.

## 4. Discussion

The CRISPR/Cas9 method and its variations have in the past years developed into a widely-used tool that facilitates targeted genome editing in mammalian cells. Not only gene knockout but also targeted correction of, for example, disease mutations can be accomplished [13,14,15,16,17], and high hopes have been raised concerning the therapeutic use of these methods. In most cases, researches control the success of the targeted gene ablation by sequencing the genomic region around the gRNA target site and by demonstrating the lack of protein expression by means of Western Blot. However, only very rarely are the consequences of the gRNA-mediated approach studied at the mRNA level. We here show, using the human *FLOT1* and *AGA* genes as examples, that very benign genomic changes may have heterogeneous consequences for the splicing of the respective hnRNA product, giving rise to alternative splicing products that may produce dysfunctional proteins instead of resulting in a complete gene knockout.

**Figure 9:**
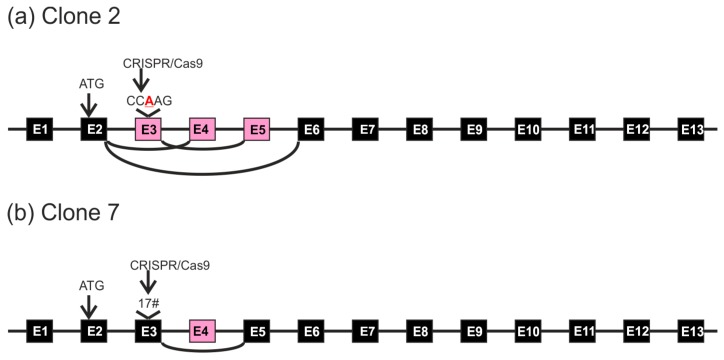
Observed genomic changes and corresponding alterations in mRNA sequences upon *FLOT1* gene knockout. (**a**) Clone 2, and (**b**) clone 7. Exons whose splicing is altered are shown in pink.

In this study, we intended to knock out the expression of flotillin-1 in HeLa cells. Our analysis at the genomic level revealed expected indel alterations in the targeted exon number 3 of the *FLOT1* gene. Western Blot analysis revealed the absence of a band at the right molecular weight position in several single-cell clones originating from different transfections. However, qPCR demonstrated that a varying percentage of flotillin-1 mRNA was still detectable in the cell clones. This prompted us to carry out an analysis of the flotillin-1 mRNA species in these cell clones. A summary of the genomic changes vs. mRNA alterations is provided for *FLOT1* gene in Figure 9. Although the respective genomic indel mutations were present in the mRNA species, they were frequently coupled with altered splicing of the nearby exons. In the mRNA species containing a single nucleotide insertion in exon 3, splicing appeared to have occurred at the normal intron-exon junctions, consistent with the unaltered consensus splice sites. However, the inclusion or exclusion of exons 3–5 apparently took place in a random manner, and we cannot exclude that yet further mRNA species may arise due to this genomic change. On the other hand, a 17 bp deletion in exon 3 was consistently associated with the lack of exon 4, implicating that different genomic alterations produced by the same gRNA can alter the splicing in various ways. Similar aberrant splicing events were detected upon knockout of the human *AGA* gene in HEK cells, demonstrating that the aberrant splicing may be a general consequence of CRISPR/Cas9-mediated genomic alterations.

There is no evidence from our own studies or in the literature that alternative splicing would be a typical feature of the *FLOT1* gene, and only a single protein product is detected in all cells we have studied so far. The only genomic alterations we detected after gRNA-mediated knockout of *FLOT1* gene in HeLa cells were associated with the target exon 3. In general, splicing of hnRNA and inclusion or exclusion of exons is frequently regulated by short, conserved sequences within the exons, known as ESE (exonic splicing enhancers) and ESS (exonic splicing silencers) that provide binding sites for splice regulator proteins [18]. Mutations that alter such ESE or ESS sites are also known to cause various diseases, such as Becker muscular dystrophy or X-linked Parkinsonism with spasticity, by altering the splicing of the respective hnRNA [19,20,21]. Therefore, it is possible that an ESE or ESS was altered by the insertion of an A in the exon 3 in *FLOT1* gene, resulting in a seemingly random inclusion of exon 3. However, why the splicing of exons 4 and 5 is also altered is not clear, since these exons exhibited no sequence alterations. It is possible that especially exon 5 may be a cassette exon that is removed upon changes in splicing of the previous exons.

Prediction of the protein products that could arise from the altered mRNA species revealed that in many cases, a frame shift due to altered splicing resulted in an early stop codon and absence of any protein product. These findings in clone 2 were also consistent with the observed reduction of the mRNA level (to about 20%), as premature stop codons frequently result in mRNA decay [22]. Thus, these altered splicing products are expected to produce a true knockout of flotillin-1 expression. However, we also observed mRNA species that were predicted to give rise to an altered protein product that would contain most of the flotillin-1 protein sequence, with an aberrant sequence included in its near-N-terminal region. However, since the mRNA levels in this clone are highly reduced, we were not able to detect such protein products by Western Blot. In clone 7, the observed mRNA species would be expected to produce an altered protein with a molecular mass of about 44 kDa, as compared to the 47 kDa WT protein. Since the mRNA level was only slightly reduced (to about 80%), we were indeed able to detect the respective aberrant protein product by Western Blot. However, this aberrant protein appears to become rapidly degraded, since proteasome inhibitors resulted in an increase in the amount of this product. Furthermore, a protein product with a higher molecular mass than the WT flotillin-1 was also detected, probably representing the ubiquitinated form. The rapid degradation of the aberrant product may be due to the fact that it is missing the palmitoylation sequence in Cys34 that is important for the membrane association of flotillin-1 [23,24]. Nevertheless, such an aberrant protein, even if it is short-lived, might exhibit dominant negative effects that obscure the interpretation of the functional consequences of the gene ablation.

Although most indel mutations are likely to produce a true knockout, we have here shown that at least in some cases, they may result in altered splicing and even expression of an aberrant protein. Therefore, the results of our study should encourage researchers who are using the CRISPR-mediated genome editing to study the consequences also at the mRNA level. Such an analysis will help to select cell clones that are true knockouts, which will give more reliable data when analyzing the functional consequences of the targeted gene ablation.

## Figures and Tables

**Table 1:** Guide RNA (gRNA) sequences ^1^ for human *FLOT1* gene.

Guide ID	Sequence 5′-3′	Target Exon
165719	ATGGTTCAGGCTGGAGCTGG	2
165720	TTTCACTTGTGGCCCAAATG	2
165721	GTACTTACCGGAGACCACCA	2
165722	GCAGAACCCTGCAAGGTGTG	3
165723	CTCCAGCCACCATGACTGGG	3

^1^ Shown are the DNA sequences cloned in the plasmid pD1301-AD.

**Table 2:** Sequences of the primers used for cloning of the genomic or cDNA fragments into a plasmid for sequencing.

Primer	Sequence 5′-3′
Flot-1-genomic-fwd	CTATAGGTACCTCCCTCTCCCTACCAACTTCCC
Flot-1-genomic-rev	CTATAGAATTCTGCAGGCAAGGGTTGAGAAGAC
Flot-1-cDNA-fwd	CTATAGGTACCATGTTTTTCACTTGTGGCCC
Flot-1-cDNA-rev	CTATAGAATTCCCGGCTGTTCTCAAAGGCTTG
AGA-pcDNA3-fwd	CTATAGGATCCATGGCGCGGAAGTCGAACTTG
AGA-pcDNA3-rev	CTATACTCGAGTTAGATGCAGTCCACTTTTTCC

**Table 3:** Sequences of the qPCR primers.

Target Gene	Primer Name	Sequence 5′-3′
Flot-1	Flot1-exon10-fwd	TATGCAGGCGGAGGCAGAAG
Flot-1	Flot1-exon12-rev	CAGTGTGATCTTATTGGCTGAA
Rpl13a	Rpl13a-fwd	CCTGGAGGAGAAGAGGAAAGAGA
Rpl13a	Rpl13a-rev	TTGAGGACCTCTGTGTATTTGTCAA
B2M	B2M-fwd	AGATGAGTATGCCTGCCGTGTG
B2M	B2M-rev	TGCGGCATCTTCAAACCTCCA
Ywhaz	Ywhaz-fwd	AGGTTGCCGCTGGTGATGAC
Ywhaz	Ywhaz-rev	GGCCAGACCCAGTCTGATAGGA

## Supplementary Materials

Supplementary materials can be accessed at: http://www.mdpi.com/2073-4409/5/4/45/s1.

## Abbreviations

The following abbreviations are used in this manuscript:

AGA:	Aspartylglucosaminidase
Cas9:	CRISPR associated sequence 9
CRISPR:	Clustered regularly interspaced short palindromic repeats
DMEM:	Dulbecco’s modified Eagle’s medium
EGFR:	Epidermal Growth Factor Receptor
ESE:	Exonic splicing enhancers
ESS:	Exonic splicing silencers
FCS:	Fetal calf serum
gRNA:	Guide RNA
hnRNA:	Heteronuclear RNA
indels:	Insertions/deletions
MAPK:	Mitogen-activated protein kinase
mRNA:	Messenger RNA
NHEJ:	Non-homologous-end-joining
shRNA:	Small hairpin RNA
siRNA:	Small interfering RNA
qPCR:	Quantitative PCR
WT:	Wildtype
