# Supplementary material for: Random Splicing of Several Exons Caused by a Single Base Change in the Target Exon of CRISPR/Cas9 Mediated Gene Knockout
Source: Cells. 2016 Dec 14;5(4):45. doi: 10.3390/cells5040045 (PMC5187529; doi:10.3390/cells5040045)
Supplement: Supplementary file 1 [file cells-05-00045-s001.pdf]

Supplementary Materials: Random Splicing of Several Exons Caused by a Single Base Change in the Target Exon of CRISPR/Cas9 Mediated Gene Knockout

Marcel Kapahnke, Antje Banning and Ritva Tikkanen

(a) WT AGA sequence (coding region, exons 1-7 only)

ATGGCGCGGAAGTCGAACTTGCCTGTGCTTCTCGTGCCGTTTCTGCTCTGCCAGGCCCTAGTGCGCTGCTCCAGC
CCTCTGCCCCTGGTCGTCAACACTTGGCCCTTTAAGAATGCAACCGAAGCAGCGTGGAGGGCATTAGCATCTGGA
GGCTCTGCCCTGGATGCAGTGGAGAGCGGCTGTGCCATGTGTGAGAGAGAGCAGTGTGACGGCTCTGTAGGCTTT
GGAGGAAGTCCTGATGAACTTGGAGAAACCACACTAGATGCCATGATCATGGATGGCACTACTATGGATGTAGGA
GCAGTAGGAGATCTCAGACGAATTAAAAATGCTATTGGTGTGGCACGGAAAGTACTGGAACATACAACACACACA
CTTTTAGTAGGAGAGTCAGCCACCACATTTGCTCAAAGTATGGGGTTTATCAATGAAGACTTATCTACCACTGCT
TCTCAAGCTCTTCATTCAGATTGGCTTGCTCGGAATTGCCAGCCAAATTATTGGAGGAATGTTATACCAGATCCC
TCAAAATACTGCGGACCCTACAAACCACCTGGTATCTTAAAGCAGGATATTCCTATCCATAAAGAAACAGAAGAT
GATCGTGGTCATGACACTATTGGCATGGTTGTAATCCATAAGACAGGACATATTGCTGCTGGTACATCTACAAAT
GGTATAAAATTCAAAATACATGGCCGTGTAGGAGACTCACCAATACCTGGAGCTGGAGCCTATGCTGACGATACT
GCAGGGGCAGCCGCAGCCACTGGGAATGGTGATATATTGATGCGCTTCCTGCCAAG ...

(b) AGA CRISPR/Cas9: exon 2, 3, 4, 5 and 6 spliced out

ATGGCGCGGAAGTCGAACTTGCCTGTGCTTCTCGTGCCGTTTCTGCTCTGCCAGGCCCTAGTGCGCTGCTCCAGC
CCTCTGCCCCTGGTCGTCAACACTTGGCCCTTTAAGAATGCAACCGAAGCAGCCGTGTAGGAGACTCACCAATAC
CTGGAGCTGGAGCCTATGCTGACGATACTGCAGGGGCAGCCGCAGCCACTGGGAATGGTGATATATTGATGCGCT
TCCTGCCAAG ...

(c) AGA CRISPR/Cas9: exon 3, 4, 5 and 6 spliced out

ATGGCGCGGAAGTCGAACTTGCCTGTGCTTCTCGTGCCGTTTCTGCTCTGCCAGGCCCTAGTGCGCTGCTCCAGC
CCTCTGCCCCTGGTCGTCAACACTTGGCCCTTTAAGAATGCAACCGAAGCAGCGTGGAGGGCATTAGCATCTGGA
GGCTCTGCCCTGGATGCAGTGGAGAGCGGCTGTGCCATGTGTGAGAGAGAGCAGTGTGACGGCTCTGTAGGCTTT
GGAGGAAGTCCTGATGAACTTGGAGAAACCACACTAGATGCCATGATCATGGATGGCCGTGTAGGAGACTCACCA
ATACCTGGAGCTGGAGCCTATGCTGACGATACTGCAGGGGCAGCCGCAGCCACTGGGAATGGTGATATATTGATG
CGCTTCCTGCCAAG ...

Figure S1. Consequences of the CRISPR/Cas9-mediated *AGA* gene knockout in HEK293T cells at mRNA level. Total RNA was isolated from the knockout clones and reverse-transcribed. The coding region of *AGA* was amplified with specific primers, cloned into a vector, and four plasmid clones were sequenced. (**a**) WT human AGA coding region (exons 1–7 only) with color-coded exons (pink: exon 1 beginning from the ATG start codon). (**b**) Clone with exons 2-6 spliced out; (**c**) Clone with exons 3–6 spliced out.

(a) WT AGA protein sequence

```
MARKSNLPVLLVPFLLCQALVRCSSPLPLVVNTWPFKNATEAAWRALASGGSALDAVESGCAMCEREQCD
GSVGFGGSPDELGETTLDAMIMDGTTMDVGAVGDLRRIKNAIGVARKVLEHTTHTLLVGESATTFAQSMG
FINEDLSTTASQALHSDWLARNCQPNYWRNVIPDPSKYCGPYKPPGILKQDIPIHKETEDDRGHDTIGMV
VIHKTGHIAAGTSTNGIKFKIHGRVGDSPIPGAGAYADDTAGAAAATGNGDILMRFLPSYQAVEYMRRGE
DPTIACQKVISRIQKHFPEFFGAVICANVTGSYGAACNKLSTFTQFSFMVYNSEKNQPTEEKVDCI
```

(b) AGA CRISPR/Cas9: exon 2, 3, 4, 5 and 6 spliced out: frame-shift and early stop

```
atggcgcggaagtcgaacttgcctgtgcttctcgtgccgtttctgctctgccaggccctagtgcgctgctccagc
 M  A  R  K  S  N  L  P  V  L  L  V  P  F  L  L  C  Q  A  L  V  R  C  S  S

cctctgcccctggtcgtcaacacttggccctttaagaatgcaaccgaagcagccgtgtaggagactcaccaatac
 P  L  P  L  V  V  N  T  W  P  F  K  N  A  T  E  A  A  V  -  E  T  H  Q  Y
```

(c) AGA CRISPR/Cas9: exons 3, 4, 5 and 6 spliced out: first 94 residues AGA, amino acids 95-232 are missing, then again AGA sequence

```
atggcgcggaagtcgaacttgcctgtgcttctcgtgccgtttctgctctgccaggccctagtgcgctgctccagc
 M  A  R  K  S  N  L  P  V  L  L  V  P  F  L  L  C  Q  A  L  V  R  C  S  S

cctctgcccctggtcgtcaacacttggccctttaagaatgcaaccgaagcagcgtggagggcattagcatctgga
 P  L  P  L  V  V  N  T  W  P  F  K  N  A  T  E  A  A  W  R  A  L  A  S  G

ggctctgccctggatgcagtggagagcggctgtgccatgtgtgagagagagcagtgtgacggctctgtaggcttt
 G  S  A  L  D  A  V  E  S  G  C  A  M  C  E  R  E  Q  C  D  G  S  V  G  F

ggaggaagtcctgatgaacttggagaaaccacactagatgccatgatcatggatggccgtgtaggagactcacca
 G  G  S  P  D  E  L  G  E  T  T  L  D  A  M  I  M  D  G  R  V  G  D  S  P

atacctggagctggagcctatgctgacgatactgcaggggcagccgcagccactgggaatggtgatatattgatg
 I  P  G  A  G  A  Y  A  D  D  T  A  G  A  A  A  A  T  G  N  G  D  I  L  M

cgcttcctgccaag...
```

Figure S2. Predicted consequences of the changes in *AGA* mRNA sequences at the protein level. ExPasy Translate tool was used to convert the sequencing data to protein sequences. (**a**) WT AGA protein sequence with the same exon color coding as in Suppl. Figure S1. The codons for the residues marked in red are compiled from the flanking exons. (**b**) Removal of exons 2–6 results in a frame shift and early stop, whereas (**c**) removal of exons 3–6 produces a putative protein containing the first 94 residues of AGA, then residues 95–232 are deleted, after which the AGA sequence continues from residue 233.
